# Sterol Extraction from Isolated Plant Plasma Membrane Vesicles Affects H^+^-ATPase Activity and H^+^-Transport

**DOI:** 10.3390/biom11121891

**Published:** 2021-12-16

**Authors:** Nikita K. Lapshin, Michail S. Piotrovskii, Marina S. Trofimova

**Affiliations:** K.A. Timiryazev Institute of Plant Physiology of the Russian Academy of Sciences (IPP RAS), 35 Botanicheskaya St., 127276 Moscow, Russia; TheNinthHost@gmail.com (N.K.L.); agro-ministr@yandex.ru (M.S.P.)

**Keywords:** *Pisum sativum* L., plasma membrane vesicles, sterols, MβCD, P-type H^+^-ATPase, H^+^-transport

## Abstract

Plasma membrane H^+^-ATPase is known to be detected in detergent-resistant sterol-enriched fractions, also called “raft” domains. Studies on H^+^-ATPase reconstituted in artificial or native membrane vesicles have shown both sterol-mediated stimulations and inhibitions of its activity. Here, using sealed isolated plasma membrane vesicles, we investigated the effects of sterol depletion in the presence of methyl-β-cyclodextrin (MβCD) on H^+^-ATPase activity. The rate of ATP-dependent ∆µH^+^ generation and the kinetic parameters of ATP hydrolysis were evaluated. We show that the relative sterols content in membrane vesicles decreased gradually after treatment with MβCD and reached approximately 40% of their initial level in 30 mM probe solution. However, changes in the hydrolytic and H^+^-transport activities of the enzyme were nonlinear. The extraction of up to 20% of the initial sterols was accompanied by strong stimulation of ATP-dependent H^+^-transport in comparison with the hydrolytic activity of enzymes. Further sterol depletion led to a significant inhibition of active proton transport with an increase in passive H^+^-leakage. The solubilization of control and sterol-depleted vesicles in the presence of dodecyl maltoside negated the differences in the kinetics parameters of ATP hydrolysis, and all samples demonstrated maximal hydrolytic activities. The mechanisms behind the sensitivity of ATP-dependent H^+^-transport to sterols in the lipid environment of plasma membrane H^+^-ATPase are discussed.

## 1. Introduction

P-type H^+^-ATPases of plant cells transform the energy of ATP hydrolysis into a transmembrane electrochemical proton gradient (∆µH^+^), in which dissipation (along with corresponding transporters) establishes the transfer of ions and metabolites across the plasma membrane [1,2,3]. P-type ATPases are a large family characterized by the presence of a conservative phosphorylated intermediate [4,5]. The phosphorylation and dephosphorylation reactions during a catalytic cycle lead to conformational changes of the enzyme accompanied by a displacement of the cytoplasmic domains, which are involved in gating the hydrophilic “pocket” inside the transmembrane area of the protein and in the transfer of the corresponding ions against their concentration gradient to the other side of the membrane [5,6,7].

The plasma membrane H^+^-ATPase of plant cells is characterized by the presence of the so-called “activated state”, which occurs when autoinhibition is lost and the C-terminal domain of the protein is involved [8,9]. This state is distinguished by a change in the H^+^/ATP coupling ratio in favor of a significant increase in proton pumping compared with ATP hydrolysis [10,11]. Nevertheless, the kinetic parameters of the enzyme can also vary and can be characterized by an increase in *V_max_* and affinity for ATP as well as a shift in the optimal pH of the enzyme to the more alkaline region, indicating an increased affinity for protons. Such effects are most clearly manifested in the presence of fusicoccin, which binds adaptor 14-3-3 proteins and the phosphorylated C-terminal domain of the ATPase [12,13]. The same effects were observed by limited trypsinolysis of ATPase with partial removal of the C-terminal domain, leading to irreversible activation of the enzyme [14]. Therefore, phosphorylation can be considered a universal regulator of plant H^+^-ATPase activity, which is associated with both an increase and a decrease in its activity depending on which amino acid residues of the C-terminal domain are the target for protein kinases [8,9].

Lysophospholipids are another important mechanism that can regulate the activity of H^+^-ATPase with the participation of the N- and C-terminal domains [15]. In the 90s experiments considering the solubilization of plasma membranes in the presence of detergents (or surfactants) showed that membrane suspension contained vesicles with different orientations of the plasma membrane surfaces (right-side-out and inside-out), i.e., 50% of these vesicles were latent in terms of access to ATP molecules [16]. The addition of non-ionic detergents that did not disrupt membrane protein conformation approximately doubled the specific ATPase activity of plasma membranes [15,17]. However, the presence of lysophospholipids showed an even stronger effect with a significant increase in *V_max_*, indicating the specific mechanism of the enzyme activation that was not associated with phosphorylation of the penultimate threonine in the C-terminal domain [15].

Since the transmembrane domains of the proteins are surrounded by lipids, such protein−lipid interactions can be considered an important condition for membrane functionality [18,19] and can occur in at least three ways: (1) due to general lipid−protein interactions, when the so-called annular lipids affect the conformational and lateral mobility of the protein generally; (2) due to specific contacts when lipids are incorporated into a protein molecule (possibly during its ER translation) and are inserted between transmembrane domains; and (3) due to the presence of specific lipid-binding motifs in proteins [20,21,22,23]. Nevertheless, local differences are present in the composition, thickness, and packing of biological membranes, and the properties of the same protein may vary depending on its lipid environment and localization.

The existence of specific lipid domains enriched in sterols and sphingolipids (so-called “rafts”) was discovered in the plasma membranes of eukaryotic cells [24]. Usually, “rafts” are associated with membrane fractions that are resistant to solubilization in the presence of cold nonionic detergents [25]. The proteomic analysis of detergent-resistant plant plasma membranes reveals the presence of common resident proteins such as glycosylphosphatidylinositol-anchored proteins as well as flotilins and/or remorins, which can be considered “raft” markers [26,27,28]. Additionally, many proteins with signaling functions and transporters (for example, ATPases, PIP-aquaporins, protein kinases, etc.) were detected in such membrane compartments. During plant acclimation, the number of ATPases and PIP-aquaporins in the detergent-resistant fraction were increased [29]. Although the functional role of “rafts” as sterol-rich domains is still questionable, the assumption is that it consists of (1) increasing the content of the transport proteins in a certain location on the membrane with its subsequent oligomerization; (2) lateral compartmentation of the plasmalemma, leading to cell polarization; and (3) creating a “comfortable” lipid environment for the adjustment of membrane protein activity [24].

Sterols are important structural components of biological membranes. Plants posses more complex sterol compositions, where sitosterol, stigmasterol, campesterol, and cholesterol are major constituents of their sterol profile in contrast with animal cells (only cholesterol) or fungi (ergosterol) [30]. In addition, plants are characterized by the presence of acylated steryl glycosides in their plasma membranes as well as steryl esters, which are localized in ER and serve as storage compounds [31]. The biosynthesis of sterols as isoprenoid-derived lipids in plants, unlike other living organisms, can occur in one of two pathways, i.e., initialized in the cytosol and/or plastids [32]. The ratio between the different sterol species can change during plant ontogenesis as well as under abiotic or biotic conditions [33,34]. Mutants with disrupted sterol biosynthesis usually suffer from dwarfism and a lack of fertility, which is not always associated with a deficiency in the synthesis of plant hormones—such as brassinosteroids, where campesterol is the precursor [35,36].

Many studies have been devoted to the elucidation of the role of the lipid environment in the functional activity of P-type ATPase (including plant H^+^-ATPase) and were recently summarized in reviews [22,23,37]. However, how exactly ATPase activity is regulated by sterols is not entirely clear. Some experimental data show both activation and inhibition of ATPases depending on the sterol content in their membrane environment [38,39]. In 1997, the studies by Grandmougin-Ferjani et al. on liposomes, where partially purified H^+^-ATPase of maize was reconstituted into soybean lipids with different compositions and contents of phytosterols, showed that sensitivity to sterols was manifested by the H^+^-transporting activity of the enzyme and, to a lesser extent, by ATP hydrolysis [38]. Based on these experimental data, the authors suggested that plant H^+^-ATPase has specific sterol-binding sites.

In the present work, sterols were extracted from the H^+^-ATPase lipid environment of isolated plasma membranes from pea roots in the presence of cyclic oligosaccharide MβCD. In order to elucidate how membrane sterol depletion is involved in H^+^-ATPase activity, the kinetic parameters of ATP hydrolysis and generation of ∆µH^+^ by plasma membrane vesicles were determined simultaneously with the estimation of extracted sterols.

## 2. Materials and Methods

### 2.1. Plasma Membrane Isolation

The plasma membranes were isolated from 5-day-old roots of etiolated pea seedlings (*Pisum sativum* L., cv Alfa) hydroponically grown at 22 °C, as described in [40]. The excised roots (~50 g) were homogenized in medium (150 mL) containing 300 mM sucrose, 10 mM EDTA, 5 mM dithiothreitol, 5 mM potassium metabisulfite, 1 mM phenylmethylsulfonyl fluoride, 0.6% (*w*/*v*) polyvinylpyrrolidone, and 100 mM Tris/HCl buffer (pH 8.0). After centrifugation (10,000× *g*; 15 min), the pellet was removed, and the supernatant was precipitated at 100,000× *g* for 30 min. The pellet containing the microsomal membrane fraction was suspended in phase buffer (300 mM sucrose, 5 mM potassium phosphate buffer (pH 7.8), 3 mM KCl, and 1 mM dithiothreitol) and combined with a phase mixture containing (*w*/*w*) 6.2% dextran T500−6.2% polyethylene glycol 3500 [41]. Then, the phases were separated by centrifugation at 2500× *g* for 5 min. The upper phase, enriched with plasmalemma, was diluted with the suspension medium (300 mM sucrose, 0.5 mM EDTA, and 10 mM BTP/MES (pH 7.2) and precipitated by centrifugation at 100,000× *g* for 30 min. After centrifugation, the pellet was resuspended in the same medium and stored at −70 °C. All procedures were carried out at 4 °C. In subsequent experiments, only membranes that were thawed once were used. The membranes were solubilized with 0.05% (*v*/*v*) Triton X-100, and protein content was determined by the Bradford method, using BSA as a standard [42].

### 2.2. Analysis of Membrane Lipids

The total lipids were extracted from the plasma membranes as described by Bligh and Dyer [43]. The lipid fraction in chloroform was separated on two-dimensional TLC Silica gel 60 (Merck, Darmstadt, Germany). For spotting, lipid extract (20–30 μg of plasma membrane protein) was used. Chromatography was performed at 20 °C using chloroform/methanol/18% ammonia (65:35:5 *v*/*v*) in the first dimension and chloroform/acetone/methanol/acetic acid/water (100:40:20:30:10 *v*/*v*) in the second dimension [44]. After separation, the plates were dried, sprayed with 25% sulfuric acid in 50% methanol, and then heated at 120 °C. Spots were identified using soybean phospholipids, phytosphingosine, and stigmasterol as the standards.

### 2.3. Sterol Extraction

For sterol extraction, plasmalemma was mixed with the suspension medium supplemented with 5–30 mM MβCD (Sigma, St. Louis, MO, USA) at a final concentration of 50–100 μg of protein mL^−1^. After 30 min of incubation at 4 °C, MβCD and its complexes with sterols were removed by sedimentation of the treated vesicles by means of centrifugation (100,000× *g* for 60 min); then, the pellet was transferred to a fresh suspension medium. The plasma membrane sterol content before and after treatment with MβCD was assessed using the Amplex Red Cholesterol Assay Kit (Invitrogen, Eugene, OR, USA) according to the manufacturer’s protocols. For all measurements, ~10 μg of plasmalemma was added to the sample buffer containing 0.1 M potassium phosphate (pH 7.4), 0.05 M NaCl, 5 mM cholic acid, and 0.1% Triton X-100. Cholesterol solutions (0.25–8 μg mL^−1^) were used as a standard. After incubation with the enzyme mixture at 37 °C for 30 min, the resorufin fluorescence intensity (Ex = 550 nm; Em = 585 nm) was recorded on a Hitachi 850 spectrofluorimeter (Japan).

### 2.4. Vesicle Size

The sizes of the membrane vesicles were determined with the dynamic light scattering method using a Photocor Compact Z particle analyzer (Photocor, Moscow, Russia). For measurements, a plasma membrane (200 μg mL^−1^) in the medium containing 100 mM sucrose, 1 mM MgSO_4_, and 10 mM BTP/MES (pH 7.2) was mixed with an equal volume of the same medium in the presence or absence of 10 mM MβCD and placed immediately into the device’s measuring chamber. The histogram of the particle size distribution was obtained after processing the measured correlation function using the DynaLS Photocor software.

### 2.5. ATP Hydrolysis

The enzymatic activity of the plasma membrane H^+^-ATPase was evaluated using a coupled ATP-regenerating system by recording the kinetics of NADH oxidation at 340 nm [45]. Vesicles (25–50 µg protein mL^−1^) were added to the solution, containing 50 mM KCl, 0.25 mM NADH, 1 mM phosphoenolpyruvate, 2.5 µL mL^−1^ mixture of pyruvate kinase and lactate dehydrogenase enzymes (Sigma, St. Louis, MO, USA), 5 mM MgSO_4_, and 10 mM MES/BTP (pH 6.5). The hydrolysis reaction was initiated by adding ATP at final concentrations from 0.1 to 4 mM. The measurements for each sample were carried out simultaneously at different ATP concentrations in a volume of 250 μL for at least 3 min using an 8-position micro-multi cell (Shimadzu UV-2700). The molar extinction of NADH used for calculations was 6.2 mM^−1^ cm^−1^. The kinetic parameters of ATP hydrolysis were estimated by approximating the experimental data with the hyperbolic model *V_o_* = *V_max_* [ATP]/([ATP] + *K_M_*) in the nonlinear regression mode of the SigmaPlot 12 software.

### 2.6. ATP-Dependent ∆pH and ∆Ψ Generation

In order to evaluate the formation of an ATP-dependent transmembrane proton gradient (∆pH), a decrease in the absorbance of acridine orange during the acidification of the vesicle lumen was recorded on a Hitachi 557 spectrophotometer using the dual-wavelength measurement mode (492–540 nm) [46]. Membrane vesicles (25–50 μg^−1^ protein mL^−1^) were mixed with a medium containing 300 mM sucrose, 50 mM KCl, 10 mM MES/BTP (pH 6.5), 6 µM acridine orange, and 1 mM ATP, and then, the reaction was initiated with 2 mM MgSO_4_. The ATP-dependent ∆pH was dissipated by adding gramicidin D (2 µM) into the spectrophotometer cell. The formation of an electric potential across the plasma membrane was detected by measuring the absorbance changes of oxonol VI [47]. The working solution contained 300 mM sucrose, 10 mM MES/BTP (pH 6.5), 2 μM oxonol VI, 1 mM ATP, and membrane vesicles (25–50 μg protein mL^−1^). The reaction of ATP hydrolysis started with 2 mM MgSO_4_. The experiments were carried out in dual-wavelength mode (580–620 nm) on a Hitachi 557 spectrophotometer. Dissipation of ∆Ψ was observed by adding 50 mM KCl into the spectrophotometer cell, thereby converting the electrical component of ∆µH^+^ into a chemical one.

### 2.7. Passive H^+^-Permeability

Passive proton leakage was studied using the method of artificial generation of the pH gradient by loading membrane vesicles with (NH_4_)_2_SO_4_ [46,48]. In the initial step, a suspension (1 mg protein mL^−1^) was mixed with 250 mM of (NH_4_)_2_SO_4_, and after a few minutes of incubation, 50 μL of these vesicles were added to 2 mL of working solution, containing 300 mM sucrose, 50 mM KCl, 10 mM MES/BTP (pH 6.5), and 6 μM acridine orange. Then, changes in the absorbance of the dye were recorded in the dual-wavelength mode (492–540 nm). The formation of the pH gradient occurred when the concentration of NH_3_ became equal on both sides of the vesicular membrane since the permeability of (SO_4_)^2−^ was significantly lower. After a decrease in the acridine orange difference absorbance, various MβCD concentrations were added directly to the loaded vesicles. The rate of ∆pH dissipation in this system could indicate the passive proton permeability of the plasmalemma. However, the transfer of protons along their concentration gradient could occur with the participation of membrane transporters, which may be sensitive to the sterol content in the plasma membrane. In order to evaluate which of the processes is involved in the disturbance of ∆pH (i.e., cotransport with anions and/or countertransport with cations), some experiments were carried out with equimolar replacement of KCl by cholineCl as well as by adding valinomycin into the measuring cuvette at the final concentration of 50 nM.

### 2.8. Detergent-Resistant Plasma Membrane Fractions

Plasmalemma microdomains enriched with sterols are characterized by resistance to solubilization by non-ionic detergents at 4 °C [26,27,28]. To separate detergent-resistant membranes (DRM) from the solubilized membrane fraction, floatation was performed in a discontinuous (30%–20%–0%) density gradient of iodixanol or OptiPrep (Sigma, St. Louis, MO, USA) [40]. To create a three-step gradient, five volumes of an initially 60% iodixanol solution were diluted with one volume of 6X suspension medium to obtain a 50% working solution. After, 50% solution was mixed with either the solubilized plasma membrane or suspension medium to create the gradient steps. Plasma membranes (~600 μg) were solubilized in the presence of 1% Triton X-100 for 30 min at 4 °C at a detergent/protein (*w/w*) ratio of 15:1. Then, 0.8 mL of the solubilized sample was mixed with 1.2 mL of 50% iodixanol and placed on the bottom of the test tube; 2 mL of the 20% solution and 0.5 mL of the suspension medium were layered on top. Then, the solubilized membranes were separated by centrifugation for 2 h at 100,000× *g* in a SW55Ti rotor (Beckman, Brea, CA, USA).

After centrifugation, four fractions (1 mL each) were collected from the top to the bottom of the test tube and then were used for Western blot analysis of the H^+^-ATPase and sterol content. Fractions 1 and 2, with densities of 1.08 and 1.13 g cm^−3^, contained trace amounts of protein and were not used further in the analysis. Fractions 3 and 4 (with densities of 1.15 and 1.18 g cm^−3^, respectively) contained a sterol-rich detergent-resistant fraction and solubilized proteins. The density of OptiPrep solutions was estimated by measuring the refractive index and comparing it with the manufacturer’s tabulated values.

### 2.9. Western Blot Analysis

In order to assess how the H^+^ ATPase is distributed between sterol-rich and other plasma membrane domains, proteins from fractions 3 and 4 were precipitated by 10% TCA. The precipitates were first washed twice with 1 M Tris until neutral pH and then solubilized in the presence of 2% SDS. The protein content was determined using the Bicinchoninic Acid Kit for Protein Determination (Sigma, St. Louis, MO, USA). Before electrophoretic separation according to Laemmli, the samples were mixed with 2X sample buffer containing 1X SDS [49]. Denaturation was carried out at 56 °C for 30 min. The samples were separated with 10% SDS-PAGE in a Mini-Protean III Cell (Bio-Rad, Hercules, CA, USA) at 200 V for 1 h. After electrophoresis, the proteins were transferred onto a nitrocellulose membrane by a semi-dry method according to Towbin [50] using a Trans-Blot SD cell (Bio-Rad, USA). The transfer time was 2 h at a current density of 2 mA cm^−2^. Primary antibodies AS07 260 (Agrisera, Vännäs, Sweden) and secondary fluorescein-labeled antibodies (Medgamal, Moscow, Russia) were used for immunodetection. The blots were scanned on a Typhoon Trio Plus (GE Healthcare, Chicago, IL, USA).

### 2.10. Statistical Analysis

Data were analyzed using one-way analysis of variance (ANOVA) in Sigma Plot software. All results are presented in the form of mean  ±  standard deviation (SD) from three to five independent experiments. A difference was considered statistically significant when *p* ≤  0.05.

## 3. Results

### 3.1. Plasma Membrane Lipids and Sterol Extraction

**Figure 1:**
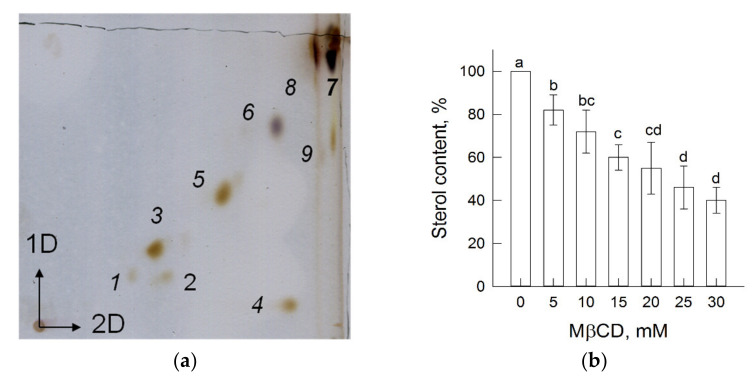
Two-dimensional thin layer chromatogram of total lipids (**a**) and sterol content (**b**) of plasma membrane vesicles treated with different MβCD concentrations. Arrows in (**a**) indicate the first and the second directions of separation. The numbers correspond to *1*—phosphatidylinositol; *2*—phosphatidylserine; *3*—phosphatidylcholine; *4*—phosphatidic acid; *5*—phosphatidylethanolamine; *6*–cerebrosides; *7*, *8*—sterols (steryl glycosides and acylated steryl glycosides); and *9*—free fatty acids. The bars in (**b**) represent mean ± SD of two to five independent biological replicates. The initial level of sterol content (85.6 ± 8.6 µg mg^−1^ protein) corresponds to 100%. Means with different letters represent significant differences (*p* < 0.05) by Tukey’s test.

Plasmalemma obtained through the partitioning of microsomal membranes in an aqueous two-phase polymer system contains all of the main classes of membrane lipids: phospholipids (*1–3*, *5*), cerebrosides (*6*), and sterols (including steryl glycosides and acyl steryl glycosides (*7*, *8*)) as well as minor amounts of phosphatidic acid (*4*) and free fatty acids (*9*) (Figure 1a). The chromatogram illustrates that the ratio between phospholipids and sterols in the plasma membrane is close to 1, as was shown in [44].

The MβCD compound is a cyclic water-soluble oligosaccharide and can extract sterols from the lipid bilayer by their diffusion from the membrane into the inner hydrophobic cavity of the oligosaccharide [51]. In order to evaluate the amount of sterols that was extracted, vesicles were precipitated by centrifugation in solutions containing different concentrations of MβCD, and then, the residual membrane sterol content was determined. The results in (Figure 1b) show the plasma membrane sterol content after MβCD treatment. The initial sterol content of isolated plasmalemma samples was 85.6 ± 8.6 μg mg^−1^ protein and gradually decreased after treatment, reaching almost 50% of the original level upon incubation of 100 μg protein mL^−1^ with 30 mM MβCD, as was shown in [52].

Considering that the diffusion of sterols between the lipid bilayer and the hydrophobic cavities of the oligosaccharide can occur in both directions, in the first series of experiments used to assess the H^+^-transporting and hydrolytic activities of the H^+^-ATPase, MβCD was directly added to the samples.

### 3.2. MβCD and H^+^-Pumping

**Figure 2:**
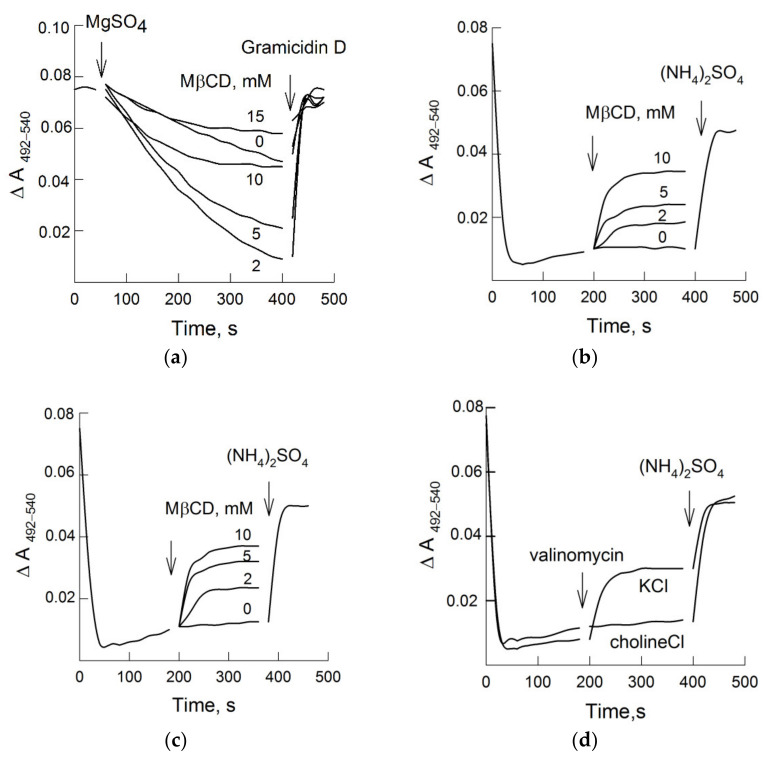
Effects of different MβCD concentrations on ATP-dependent (**a**) and passive (**b**–**d**) H^+^-transport. Plasma membranes were added into a medium with 50 mM KCl and MβCD (**a**). In (**b**–**d**), vesicles were preliminary loaded with (NH_4_)_2_SO_4_ and then placed into measuring medium supplemented with 50 mM KCl (**b**) or 50 mM cholineCl (**c**). After a decrease in the absorbance of acridine orange, MβCD (**b**,**c**) or 50 nM valinomycin (**d**) was added. ATP-dependent ∆pH was dissipated by 2 µM Gramicidin D; the artificial pH gradient was disrupted by the addition of 10 mM (NH_4_)_2_SO_4_. Representative kinetics curves are presented.

Figure 2a presents the kinetics of transmembrane ∆pH generation across the vesicular membrane. The reaction started only after the addition of Mg^2+^ (which forms a complex of MgATP, a substrate for P-type ATPases) and was accompanied by a decrease in the difference absorbance of acridine orange due to acidification of the vesicles’ lumen. We discovered that MβCD at low concentrations significantly stimulated H^+^-pumping. In addition, as the concentration of MβCD increased, a decrease in the rate of ATP-dependent H^+^-transport was observed (Figure 2a). Despite sealed plasma membrane vesicles being a relatively simplified model, at least three possible mechanisms related to the effects of sterol extraction on active proton transport were experimentally studied. First, on the same vesicle suspension and corresponding MβCD concentrations, the hydrolytic activity of H^+^-ATPase was evaluated; second, the passive permeability of the plasma membrane for protons was estimated; and, lastly, the sizes of the vesicles were measured.

### 3.3. ATP Hydrolysis

An analysis of the kinetic parameters of ATP hydrolysis coupled with NADH oxidation showed that the enzymatic activity of H^+^-ATPase was practically unaffected by sterol depletion. The *K_M_* values did not change at all, while the rate of ATP hydrolysis slightly increased. In addition, the MβCD concentrations that had a stimulating effect on hydrolysis and H^+^-transport did not coincide (Table 1), i.e., the extraction of up to 10% of membrane sterols increased the H^+^-transporting activity but did not affect the ATP hydrolysis. Thus, it can be assumed that the significant stimulation of transport in comparison with hydrolysis might be interpreted as an alteration of the enzyme H^+^/ATP coupling ratio.

### 3.4. MβCD and Plasma Membrane H^+^-Leakage

A decrease in ATP-dependent H^+^ transport may be the result of so-called “futile” activity by the enzyme, when the passive proton permeability through the lipid bilayer and/or other membrane transporters becomes comparable with the active component of ATP hydrolysis. An assessment of the passive permeability of the plasmalemma for protons, when the vesicles’ lumen was loaded with (NH_4_)_2_SO_4_, showed that, in the absence of MβCD, a rapid drop in the absorbance of acridine orange was observed, followed by its slow recovery (Figure 2b–d).

As shown in Figure 2b, the addition of MβCD to the suspension of vesicles with a preformed transmembrane pH gradient caused a dose-dependent effect of its discharge. The observed effects of ∆pH dissipation did not depend on the nature of the added cation and were identical in media containing K^+^ or choline^+^ (Figure 2c). However, the stimulation effects of K^+^ on proton leakage were clearly manifested in the presence of the selective ionophore valinomycin (Figure 2d). These data indicate that the increase in H^+^-permeability in response to MβCD was most likely not associated with the activity of cation/proton exchange, since it occurred in the absence of K^+^ (Figure 2c). Interestingly, the generation of a potential with “+” inside the vesicles’ lumen by valinomycin also initiated proton leakage (Figure 2d). In this case, the following sequential and interrelated process of proton leakage enhancement in the presence of MβCD can be considered: first, Cl^−^ must enter the vesicles’ lumen, which, in turn, triggers H^+^/anionic cotransport. Table 1 shows that passive proton permeability was almost linearly dependent on the membrane sterol content, in contrast with H^+^-pumping, the changes of which had a transitory characteristic.

### 3.5. Sterol Extraction and Vesicle Size

**Figure 3:**
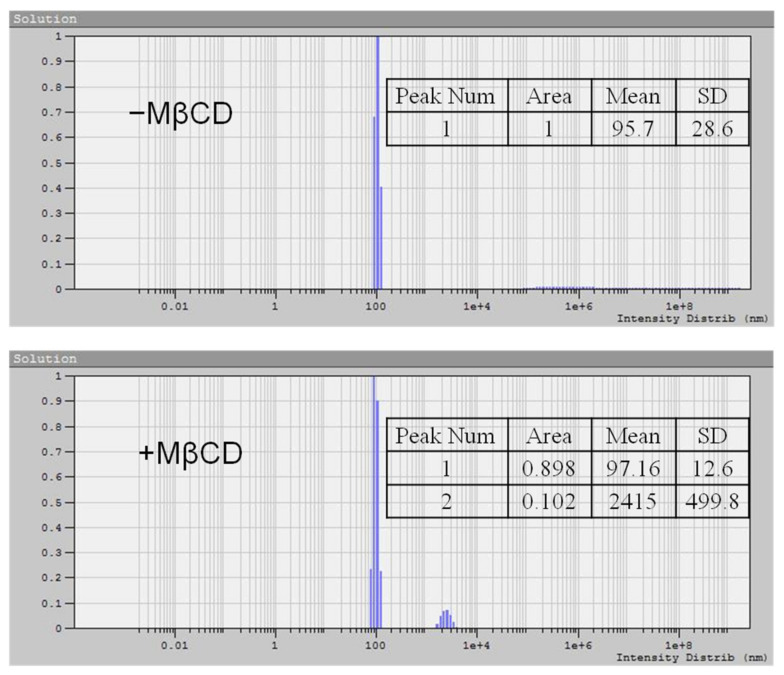
Distributions of particle sizes obtained by Dynamic Light Scattering in the absence (top) or presence (bottom) of 10 mM MβCD in vesicular suspension. Particle radiuses are represented by means ±SD.

Figure 3 shows the sizes of membrane vesicles treated in the presence of MβCD. When the suspension was incubated with MβCD, particles with sizes of more than 2 μm were detected, which correspond to aggregates of oligosaccharides with occluded sterols [51]. The proportion of such particles did not exceed 10%, although the dimensions of the light-scattering vesicle population did not change.

### 3.6. Sterols and ∆Ψ Generation

**Figure 4:**
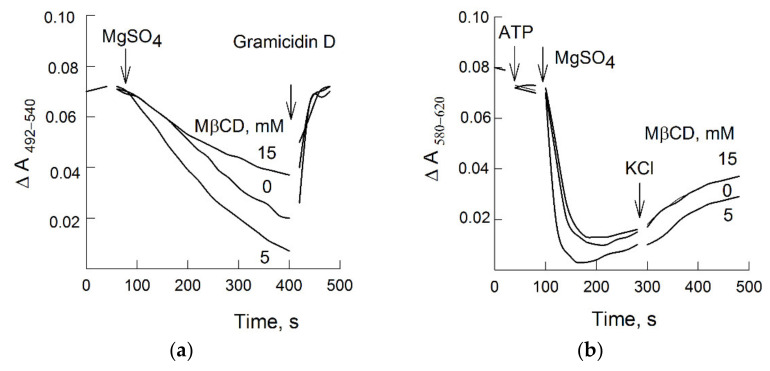
Generation of ∆pH (**a**) and ∆Ψ (**b**) by plasma membrane vesicles with different sterol contents. Sterols were extracted by treatment of plasma membranes in the presence of different MβCD concentrations, and then, vesicles were washed from sterols/MβCD complexes by centrifugation. Sterol content was as shown in Table 2. ATP-dependent ∆pH and ∆Ψ were dissipated by 2 µM Gramicidin D and 50 mM KCl, respectively. Representative kinetics curves are presented.

In order to distinguish between the effects of sterol depletion on ATP hydrolysis and H^+^-transport, vesicles were washed from MβCD/sterol aggregates and used in subsequent experiments. Moreover, the potential-dependent dye oxonol VI was previously found to be able to interact with the hydrophobic cavity of the MβCD. Thus, in order to study the ability of vesicles with different sterol contents to generate an ATP-dependent potential difference, first, the plasma membranes (treated with 5 and 15 mM MβCD, respectively) were washed from MβCD/sterol complexes. All three samples, with different contents of sterols (Table 2), retained the ability to generate ∆Ψ, which manifested as the quenching of the oxonol VI difference absorbance. Anionic dye entered the vesicles by potential gradient (with “plus” inside vesicles), created by the pump activity. The results are presented in Figure 4b. Interestingly, significant differences in the ∆Ψ values between the samples were not observed, which could be a result of the limited electrical capacitance of the vesicles. The addition of KCl into the medium led to the dissipation of membrane potential in all three variants. The results of ∆pH measurements confirmed that the activation of proton transport is the major effect of sterol extraction from PM (Figure 4a). The times required for reaching the steady-state values of ∆Ψ and ∆pH were significantly different. Most likely, chlorine anion (which quenches the electric charge inside the vesicles’ lumen) acts as a counter ion and promotes the accumulation of protons. The exchange of potassium ions for protons, apparently, is not active.

### 3.7. ATP Hydrolysis in the Presence of Dodecyl Maltoside (DDM)

**Figure 5:**
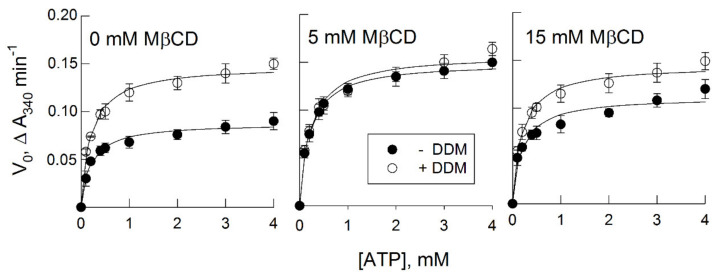
Hydrolysis of ATP by plasma membrane vesicles with different sterol contents in the presence or absence of dodecyl maltoside (DDM). Sterols were extracted by treatment of plasma membranes in the presence of different MβCD concentrations, and then, vesicles were washed from sterols/MβCD complexes by centrifugation. The initial velocities (*V*_0_) represent means ± SD. Values of *K_M_* and *V_max_* are presented in Table 2.

The same vesicles were tested for ATP hydrolysis. The *K_M_* values were practically identical among all samples (Table 2); however, *V_max_* was noticeably higher for vesicles with enhanced proton transport compared with the control (Figure 4a). After the freezing and thawing procedure, the plasma membrane vesicles acquire a 50/50 orientation, i.e., the number of right-side-out and inside-out vesicles coincides and only half of the vesicular population has access to ATP at the membrane surface [16]. A non-ionic detergent, DDM, was previously shown to solubilize membranes while preserving H^+^-ATPase activity to maintain its conformation in its native state, to provide access to ATP for all enzyme molecules, and to gently remove bulk and annular lipids [53]. Vesicles with different sterol contents treated in the presence of DDM showed a twofold increase in ATPase activity (Figure 5), i.e., the rate of ATP hydrolysis in all samples became equal (Table 2). These data may suggest that the observed effects took place as long as the H^+^-ATPase was in its native membrane environment, and annular sterols are most likely involved in these processes.

### 3.8. H^+^-ATPase in Detergent-Resistant Fractions

The presence of sterols in the protein membrane environment is an important circumstance for its localization in sterol-rich membrane domains—so called “rafts” [24]. Therefore, the experimentally obtained detergent-resistant fractions of the plasmalemma can be associated with lipid rafts. The study of the protein composition in sphingolipid- and sterol-rich membranes makes it possible to study whether the target protein is heterogeneously localized on the membrane surface.

**Figure 6:**
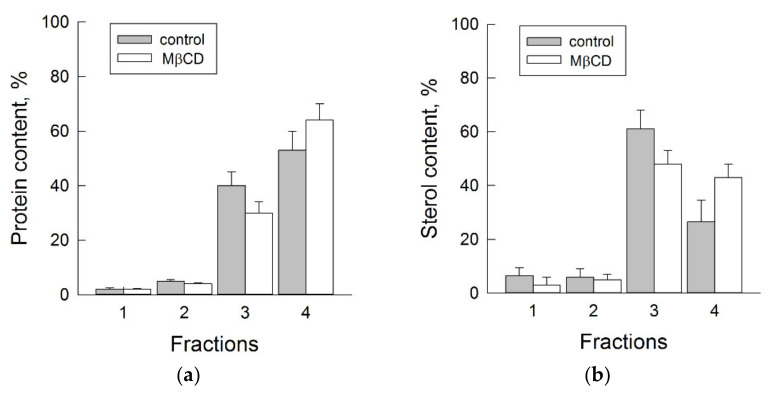
Effects of MβCD on protein (**a**) and sterol (**b**) distribution between Triton X-100-solubilized plasma membrane fractions obtained after their floatation in iodixanol density gradient. All gathered protein/sterol contents after centrifugation were taken as 100%. Fractions 1–4 corresponded to 1.079, 1.127, 1.156, and 1.185 g cm^−3^, respectively. Plasma membranes were pretreated with 10 mM MβCD before adding Triton X-100.

**Figure 7:**
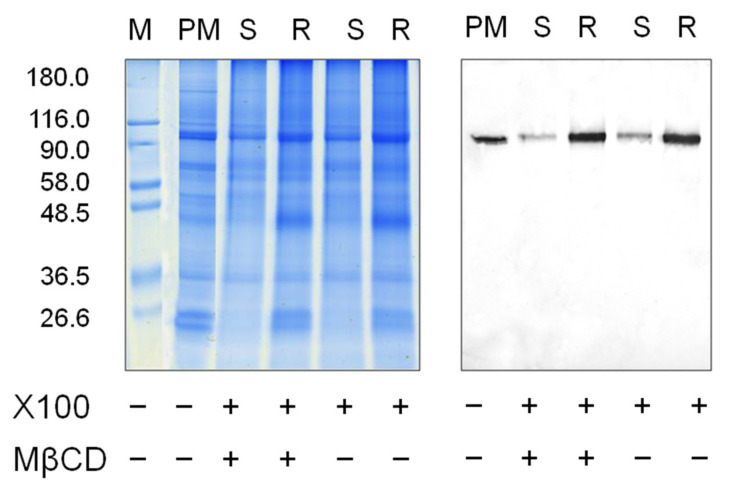
Effects of MβCD on protein distribution (left) and H^+^-ATPase content (right) between Triton X-100-resistant (R) and X-100-soluble (S) plasma membrane (PM) fractions obtained after separation in iodixanol density gradient. Plasma membranes were pretreated with 10 mM MβCD before adding Triton X-100. R and S correspond to bars 3 and 4 from Figure 6.

Figure 6 represents experimental data on plasma membrane fractions solubilized by cold Triton X-100. In this case, protein material resistant to detergent was localized in sterol-rich fraction (Figure 6a, bar 3). In addition, the fraction with the solubilized plasma membrane material was enriched in protein (Figure 6a, bar 4). Interestingly, the protein content solubilized with Triton X-100 slightly increased if the membranes were pretreated with MβCD. Apparently, sterol extraction from plasma membrane affects the ratio between soluble and non-soluble proteins in the presence of Triton X-100. However, the ratio between sterol content in the soluble (Figure 6b, bar 4) and resistant (Figure 6b, bar 3) fractions became equal. The segregation of sterols can be assumed to depend on their concentration in the lipid bilayer, and a decrease in sterol content facilitates their solubilization by Triton X-100. A Western blot analysis of the same fractions showed that, first, H^+^-ATPase was simultaneously detected in both the detergent-resistant and detergent-soluble fractions, and second, the extraction of sterols did not change the pattern of its distribution (Figure 7).

## 4. Discussion

In plant cells, the energy of transport processes in the form of ∆µH^+^, which is based on the activity of P-and V-type H^+^-ATPases, can be effective when passive diffusion of hydrogen ions across the lipid bilayer of cell membranes is limited. The presence of various molecular types of sterols in plants is also important for maintaining a low proton leakage of their membranes [54]. That study of the proton permeability of artificial bilayers with lipid compositions inherent for “rafts” (i.e., with sterols and sphingolipids) showed that, in their presence, the proton permeability can both increase and decrease and depends on the general phospholipid environment of these molecules [55]. Sterols embedded into the lipid bilayer are known to significantly modulate the physical properties of membranes, affecting the ordering of phospholipids and the motion of their acyl fatty acid chains [56]. Nevertheless, it is not entirely clear which mechanism (water “wires”, water “clusters”, or weak acids) is responsible for proton diffusion along its concentration or potential gradient [55].

Sterol extraction from the plasmalemma of pea roots in the presence of 10 mM MβCD led to an increase in passive proton permeability, which was expressed in the dissipation of an artificial pH gradient (Figure 2b,c).

The effects of sterols may have an impact on the permeability of the lipid bilayer for protons. However, this assumption, from our point of view, requires additional experiments, since the plasma membrane contains other transporters and channels, for which threshold values of the sterol content may differ from values required for the activation or inhibition of the H^+^-ATPase. Thus, phytosterols modulate the selectivity while only stigmasterol alters the voltage-dependence of the plant VDAC in the range of sterol fraction found in the outer mitochondrial membrane [57].

The main result of our work was the fact that the enhancement of ATP-dependent H^+^-transport in response to sterol extraction had a transitory nature (Figure 2a and Table 1). These results coincide with the data of Grandmougin-Ferjani et al., who showed that the H^+^-transport of liposomes with inserted H^+^-ATPase was nonlinearly dependent on the sterol content in the enzyme environment [38]. Since the effects of cholesterol, in contrast with other sterols, were most pronounced, the authors suggested that the binding sites for the interaction of sterols with the enzyme are specific.

Nevertheless, as was shown earlier in [52], in a similar concentration range, MβCD extracts different types of sterols from the plasmalemma of BY2 tobacco cells in proportion to their content and without showing any preferences. Thus, the sterol content, most likely, through a change in the physical properties of the lipid bilayer, can be assumed to affect the coupling of ATP hydrolysis with the capture and transfer of protons by H^+^-ATPase. However, our experiments showed that sterol extraction from the plasmalemma causes an increase in proton leakage, which can be explained by the MβCD effects on the anionic conductivity of the membrane (Table 1).

Specific binding sites for sterols were found in Na^+^/K^+^-ATPase [58]. The sterols of these sites are most likely non-annular lipids, and their extraction in the presence of MβCD occurred when open membrane fragments were treated with SDS. The activity of Na^+^/K^+^-ATPase sharply decreased when the cholesterol content in the membrane dropped to 30% of its initial level. The authors believe that the extraction of sterols with a content of less than 70% did not have a significant impact on the steady-state activity of the enzyme, and only when this threshold value was reached did the E2 state (by Post-Albers cycle) predominantly stabilize relative to the E1 state, which led to the inhibition of the Na^+^/K^+^-ATPase activity.

The experiments (Figure 5 and Table 2) showed that, when H^+^-ATPase from membrane vesicles with different sterol contents passed into DDM micelles (i.e., the hydrophobic environment of the enzyme changed), the kinetic parameters of ATP hydrolysis became almost identical. Sterols can be assumed to play an important role as annular lipids for the H^+^-ATPase, and a significant increase in ATP-dependent proton transport may be associated with the conformational mobility of the actuator A domain of the enzyme relative to the bilayer [6,7]. The steady-state activity of the enzyme solubilized with DDM did not depend on the sterol content even when it was decreased to 50% of its initial level before the addition of DDM.

The results on membrane solubilization in the presence of another non-ionic detergent Triton X100 showed that H^+^-ATPases are heterogeneously distributed in plasma membranes and are detected among proteins with different densities of surrounding sterols, which is expressed in their detection in both detergent-resistant and solubilized membrane fractions (Figure 7). Sterol extraction in the presence of MβCD equalized their distribution between the detergent-resistant and detergent-soluble fractions and increased the amount of solubilized proteins but did not change the ratio between the heterogeneous distribution of H^+^-ATPases (Figure 6 and Figure 7). At least two pools of H^+^-ATPases can be assumed to be present, i.e., sterol-dependent and sterol-independent.

Thus, in [59], where a proteomic analysis of detergent-resistant fractions was performed after treatment of plasma membranes with MβCD, the authors concluded that sterol-rich membranes can contain both “constant” core components, mainly containing cell wall-related proteins and lipid-modifying activities, as well as a “dynamic” component, which includes receptor-like kinases and other signaling proteins. In addition, when detergent-resistant domains are not sensitive to MβCD, they are not part of the “raft model” but still act as membrane regions that are resistant to treatment with Triton X-100. MβCD, as was shown in experiments with giant unilamellar liposomes [60] (and was also shown in [61] using the method of multiscale molecular dynamics simulations), primarily extracts sterols from disordered bilayers. The authors believe that the contents of sterols in the ordered and disordered phases of the lipid bilayer were in equilibrium, and when the sterol content decreased, the differences between the phases slowly disappeared and the coexistence of the two phases ceased [60,61]. Taking into consideration the rapidly manifesting effects of MβCD on ATP-dependent proton transport, most likely, the sterol pool of the disordered phase is involved, which is quickly extracted by MβCD-dimers.

Could the extraction of sterols from the plasma membrane make physiological sense? First, how sterols are distributed between the outer and inner leaflets of the plasma membrane is not clear. Apparently, this question concerns how sterols enter the plasma membrane, i.e., via vesicular transport from the ER and Golgi complex [62] or via lipid transfer proteins [63]. Sterol-binding activity is enacted by oomycete peptides—elicitins, such as cryptogenin, which extracts sterols from the outer leaflet of the plasma membrane and then interacts with the corresponding receptor in sterol-bound form [64]. Based on our experiments, sterols of the inner cytoplasmic leaflet of the plasma membrane, which are targeted by MβCD, end up there without the participation of vesicular transport but via sterol-transferring proteins. However, the most prominent example can be considered to be growing pollen tubes, where in the apical region, the high concentration of sterols in the apex and the high density of H^+^-ATPases in the subapical region have a clear distinction [65].

## Figures and Tables

**Table 1:** Effects of different MβCD concentrations on kinetics parameters of ATP hydrolysis, rates of ATP-dependent H^+^-pumping, and passive H^+^-permeability of plasma membrane vesicles. Measurements were carried out in a medium supplemented with 50 mM KCl. Data (mean ± SD) from three independent membrane isolations are presented. * Indicates significant (*p* < 0.05) differences with control membranes.

MβCD	*K_M_*	*V_max_*	H^+^-Pumping	H^+^-Permeability
mM	mM	µmol ATP mg^−1^ Protein min^−1^	∆A 10^3^ min^−1^	∆A 10^3^ min^−1^
0	0.220 ± 0.07	0.82 ± 0.01	8 ± 3	2 ± 1
2	0.198 ± 0.05	0.83 ± 0.03	25 ± 8 *	7 ± 3 *
5	0.188 ± 0.04	1.18 ± 0.04	21 ± 6 *	11 ± 5 *
10	0.204 ± 0.08	1.32 ± 0.05 *	14 ± 4	14 ± 5 *
15	0.192 ± 0.07	0.70 ± 0.02	8 ± 4	n.d.

**Table 2:** Kinetic parameters of ATP hydrolysis by plasma membrane vesicles with different sterol contents in the absence or presence of dodecyl maltoside (DDM). Sterols were extracted by treatment of plasma membranes in the presence of different MβCD concentrations, and then, vesicles were washed from sterols/MβCD complexes by centrifugation. Before ATP, 10% DDM was added into a measuring cell at a detergent/protein ratio of 7:1 (*w/w*). Data from two independent sterol extractions are presented. * indicates significant (*p* < 0.05) differences compared with the initial plasma membranes.

MβCD	Sterol Content	*K_M_*		*V_max_*	
mM	μg mg^−1^ of Protein	mM		µmol ATP mg^−1^ Protein min^−1^	
		−DDM	+DDM	−DDM	+DDM
0	85.6 ± 8.6	0.196 ± 0.03	0.201 ± 0.02	0.73 ± 0.02	1.19 ± 0.03 *
5	65.5 ± 9.4	0.200 ± 0.04	0.219 ± 0.03	1.21 ± 0.03 *	1.27 ± 0.05 *
15	38.4 ± 8.2	0.215 ± 0.03	0.197 ± 0.03	0.93 ± 0.03	1.17 ± 0.03 *

## Data Availability

The data presented in this study are contained within the article.
